# Modeling the Contact Interaction of a Pair of Antagonist Teeth through Individual Protective Mouthguards of Different Geometric Configuration

**DOI:** 10.3390/ma14237331

**Published:** 2021-11-30

**Authors:** Anna Kamenskikh, Alex G. Kuchumov, Inessa Baradina

**Affiliations:** 1 Department of Computational Mathematics, Mechanics and Biomechanics, Perm National Research Polytechnic University, 614990 Perm, Russia; anna_kamenskih@mail.ru; 2 Department of Orthopedic Dentistry and Orthodontics with the Course of Children’s Dentistry, Belarusian Medical Academy of Postgraduate Education, 220013 Minsk, Belarus; baradina@yandex.by

**Keywords:** mouthguard, occlusal contact, friction, teeth

## Abstract

This study carried out modeling of the contact between a pair of antagonist teeth with/without individual mouthguards with different geometric configurations. Comparisons of the stress–strain state of teeth interacting through a multilayer mouthguard EVA and multilayer mouthguards with an A-silicon interlayer were performed. The influence of the intermediate layer geometry of A-silicone in a multilayer mouthguard with an A-silicon interlayer on the stress–strain state of the human dentition was considered. The teeth geometry was obtained by computed tomography data and patient dental impressions. The contact 2D problem had a constant thickness, frictional contact deformation, and large deformations in the mouthguard. The strain–stress analysis of the biomechanical model was performed by elastoplastic stress–strain theory. Four geometric configurations of the mouthguard were considered within a wide range of functional loads varied from 50 to 300 N. The stress–strain distributions in a teeth pair during contact interaction at different levels of the physiological loads were obtained. The dependences of the maximum level of stress intensity and the plastic deformation intensity were established, and the contact parameters near the occlusion zone were considered. It was found that when using a multilayer mouthguard with an A-silicone interlayer, there is a significant decrease in the stress intensity level in the hard tissues of the teeth, more than eight and four times for the teeth of the upper and lower teeth, respectively.

## 1. Introduction

### 1.1. Problem Context

The dentofacial system is vital as its elements maintain various physiological processes, such as respiration, digestion and speech [1]. Dental biomechanics issues have increased in number over the past decade. These issues include the study of the heterogeneity of dental tissues [2], modeling the teeth stress–strain state under orthodontic loads [3], orthodontics problems to correct the occlusion [4] and numerical simulation of contact between teeth and dental implants or mouthguards [5]. Furthermore, the condition of the dentofacial system has a significant influence on physiological processes due to tooth injuries during sports and hard physical labor as well psychoemotional stress [6,7].

Today, one of the most effective ways to avoid dental injuries is the use of a mouthguard [8]. As various mouthguard designs exist on the market, there is a need for computer modeling of biomechanical behavior, both of the structures themselves and of the materials from which they are made [9].

Research has been carried out on the following:

- Biomechanical analysis of the effect of the properties of mouthguard materials on the deformation behavior of dental hard tissues [10,11];
- Mathematical modeling of the contact interaction of mouthguards of different geometric configurations with a wide range of physiological loads [12,13];
- A comprehensive interdisciplinary study of the patterns of change in the stress–strain state of the human dentofacial system when using mouthguards [14];
- The influence of the geometric configurations of the mouthguards on the stress–strain state [15], etc.

Currently, there is a particular interest in studying the structural features of mouthguards on the elements of the dentofacial system in order to identify qualitative and quantitative patterns of deformation behavior of teeth.

### 1.2. Research Objectives

The study objectives were formulated to evaluate the practical application of multilayer Eva mouthguards with an A-silicone interlayer. The research objectives are:

- To solve the problem of deformation of teeth during occlusion for a specific clinical case, with/without mouthguards;
- To model the frictional contact in the area of teeth occlusion;
- To use an elastoplastic model of behavior on the base material of the EVA mouthguards;
- To model various configurations of mouthguards for a clinical case;
- To analyze the influence of the geometry and thickness of the A-silicone layer on the system “mouthguard–teeth”.

### 1.3. Problem Description

Analysis of the influence of the geometric configuration of protective mouthguards on their performance during teeth contact is presented in this study. In this case, the load of the jaw compression is considered as the indentation force with consideration of the friction between the contacting surfaces.

The paper presents a comparative analysis of the deformation behavior of a pair of antagonist teeth during frictional contact interaction through mouthguards of different geometric configurations.

**Figure 1:**
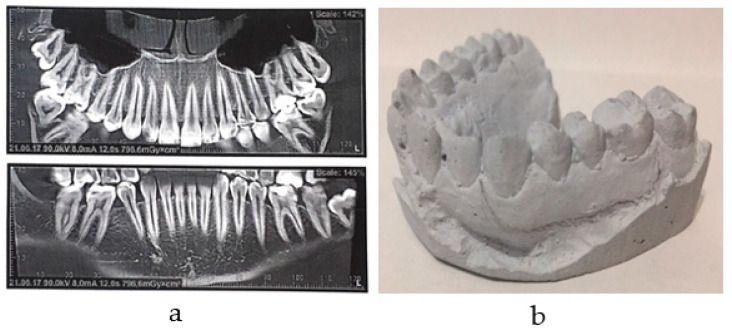
Geometry of the clinical cases: (**a**) CT image; (**b**) the gypsum model of an upper jaw.

The task was carried out on the basis of the clinical case data. The patients practiced sports professionally. Figure 1 shows the components of a biomechanical unit for one of the clinical cases: the upper jaw cast and CT (Computer tomography) image.

Improving the performance of protective mouthguards by introducing additional layers of materials has been considered by many scientific groups [16,17,18]. In [16], the novel idea of introducing an additional layer of A-silicone into the design of an ethylene–vinyl acetate (EVA) mouthguard was proposed.

The influence of the geometric characteristics of mouthguards on the stress–strain state of the hard tissues was carried out in Reference [19]. When analyzing the results, it was found that the geometric configuration of the A-silicone interlayer of the mouthguard has a significant effect on the deformation behavior of the dentition. However, in that work, the canonical geometries of the teeth were considered in contact through an individually adaptable mouthguard.

## 2. Materials and Methods

### 2.1. Design of the Experiment

In this work, an attempt was made to analyze the influence of the geometric configuration and the thickness of the interlayer on the deformation behavior of the elements of the dentition. The peculiarity of the models is the use of data on the geometry of teeth for a real clinical case. To fully evaluate the effectiveness of a multilayer mouthguard with an A-silicone interlayer, the contact of a pair of antagonist teeth was simulated for a clinical case (Figure 1) with and without the multilayer EVA of an individual protective mouthguard.

**Figure 2:**
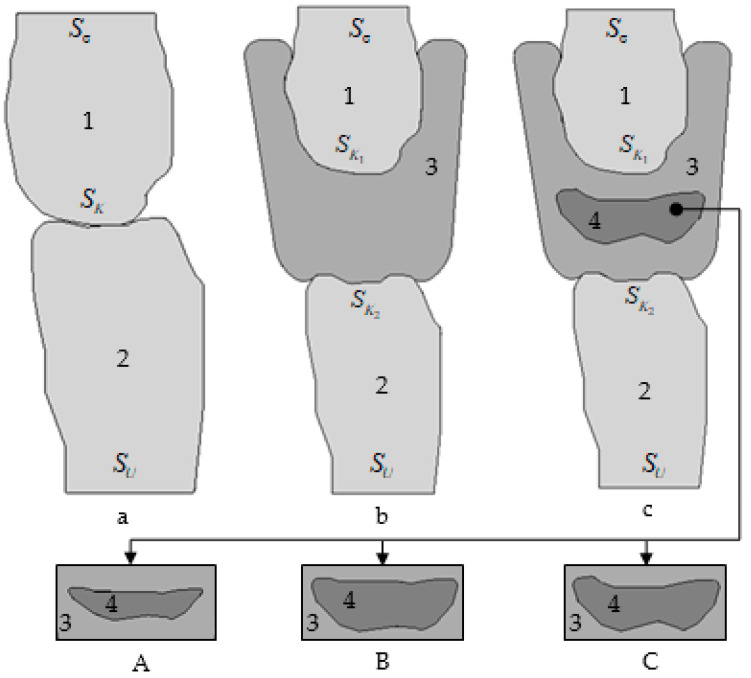
Numerical scheme of the contact between upper (1) and lower (2) teeth with and without a mouthguard: (**a**) case a without mouthguard; (**b**) case b with a multilayer EVA (3) mouthguard; (**c**) case c with a multilayer EVA (3) mouthguard with an A-silicone interlayer (4) of different geometries and thicknesses (**A**–**C**). ($S_{\sigma }$ is the boundary where the loading is applied; $S_{K}$ is the boundary where the contact occurs (for multilayered mouthguards $S_{K}{=S}_{K_{1}}\cup S_{K_{2}}$); $S_{U}$ is the boundary where displacements are set).

Five numerical models of the contact of a pair of teeth, with and without protective mouthguards of different geometries (Figure 2), were analyzed.

The geometric configuration of the teeth was based on clinical case data (Figure 1). Mouthguard fit of the teeth geometry and frictional contact were considered. In case a, frictional contact was taken into account in the area of teeth-antagonists occlusion. In cases b, c-A, c-B, c-C, antagonist teeth were in contact with mouthguards.

The maximum thickness of multilayer mouthguards in the area of teeth occlusion was about 7 mm. All multilayer mouthguards were based on EVA layers. Contact interaction between EVA layers was not considered (modeled as a solid). Multilayer mouthguards with a layer of A-silicone were modeled within the framework of the frictional contact between the layers of EVA and A-silicone.

To analyze the influence of mouthguard on the teeth deformation under contact, three variants of the A-silicone interlayer were considered (Figure 2):

- Case c-A—the thickness of the A-silicone interlayer in the occlusion region was 1.6–1.9 mm (22.8–27.1% of the maximum thickness of the mouthguard);
- Case c-B—the thickness of the A-silicone interlayer in the occlusion region was 2.9–3.2 mm (41.4–45.7% of the maximum thickness of the mouthguard);
- Case c-C—the thickness of the A-silicone interlayer in the occlusion region was 2.2–3.2 mm (31.4–45.7% of the maximum thickness of the mouthguard).

For the first two variants of the geometric configuration of the A-silicone interlayer, a slight change in thickness in the occlusion region of 10–15% is characteristic. The third variant of the interlayer has a more significant change in thickness in the occlusion region (more than 30%). It should also be noted that the interlayer thickness was adjusted to the teeth geometry: for the area where the tooth has a smaller contact area, the interlayer was selected to be the thinnest and vice versa.

### 2.2. Mechanical Properties of the Mouthguard Components

A multilayered mouthguard construction was studied. The mouthguard was from EVA (Drufosoft, Dreve, Germany) with an embedded layer of A-silicone (UfiGelP, Voco, Germany). UfiGelP is a base paste and a catalyst paste, which can be mixed in certain ratios (in this case 1:1). Once cured, UfiGelP is a highly elastic polymer material. The proportion of base and catalyst pastes in the manufacture of the splice tray was selected empirically. Before solidification, the material is soft enough and easily adjusts to the required shape. That allows you to form a layer of A-silicone in the mouthguard. The properties of these materials were obtained from experimental studies performed by the research team from Perm National Research Polytechnic University, Perm State Medical University and Perm State National Research University [16,20]. The Young’s modulus *E* and Poisson’s coefficient v are *E* = 17.3 MPa, v=0.46 (EVA) and E=0.3 MPa, v=0.49 (A-silicone). It was shown that EVA exhibits elastoplastic properties. The experimental plots are presented in Reference [20]. To describe the EVA mechanical properties, the deformation theory of elastoplasticity was chosen. A-silicone was shown to be an elastic material.

### 2.3. Loading and Boundary Conditions

The mathematical problem statement is described in Reference [20] and includes equilibrium equations, physical and geometric relations, as well as contact boundary conditions. When realizing the problem, the mathematical formulation was supplemented by considering the possibility of the appearance of large deformations in the EVA. The task was implemented as a 2D problem. The mathematical formulation of the problem was supplemented by boundary conditions: a constant load varying from 50 to 300 N (indentation force) was applied at the boundary Sσ; at the boundary S_u_, movement along the vertical coordinate *y* was prohibited.

### 2.4. Numerical Finite Element (FE) Solution and Convergence

The numerical schemes were implemented in the ANSYS software package (version 11.0, ANSYS Inc., Canonsburg, PA, USA), by the finite element method using quadrangular plane finite elements with Lagrangian approximation and two degrees of freedom at each node. The basic procedures for constructing FE models are based on the use of the Galerkin method procedure with the choice of basic functions with a compact support by the finite element method.

The analysis of the convergence of the results of the numerical solution of the problem of contact interaction of a pair of teeth with and without a protective mouthguard from the degree of discretization of the system was carried out in an earlier paper [21]. It was found that a finite element mesh with a gradient concentration of elements to the contact zones provides an optimal solution to the problem in terms of accuracy and computation time: the maximum element size is 0.25 mm, the minimum element size is approximately 4 times less than the maximum one. The finite element subdivision of the biomechanical units considered in this work was performed similarly to the previously selected mesh.

## 3. Results

The influence of the mouthguard geometry on the stress–strain state and the parameters of the occlusion region was considered in our findings.

**Figure 3:**
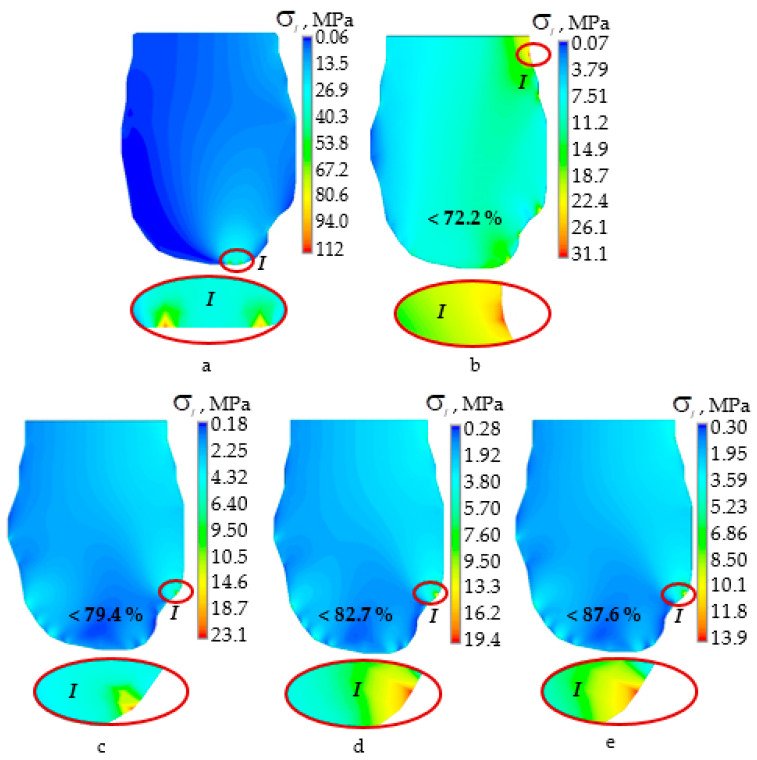
The stress intensity in the upper dentition tooth at 250 N: (**a**) case a; (**b**) case b; (**c**) case c-A; (**d**) case c-B; (**e**) case c-C; I—zone of maximum stress intensity.

**Figure 4:**
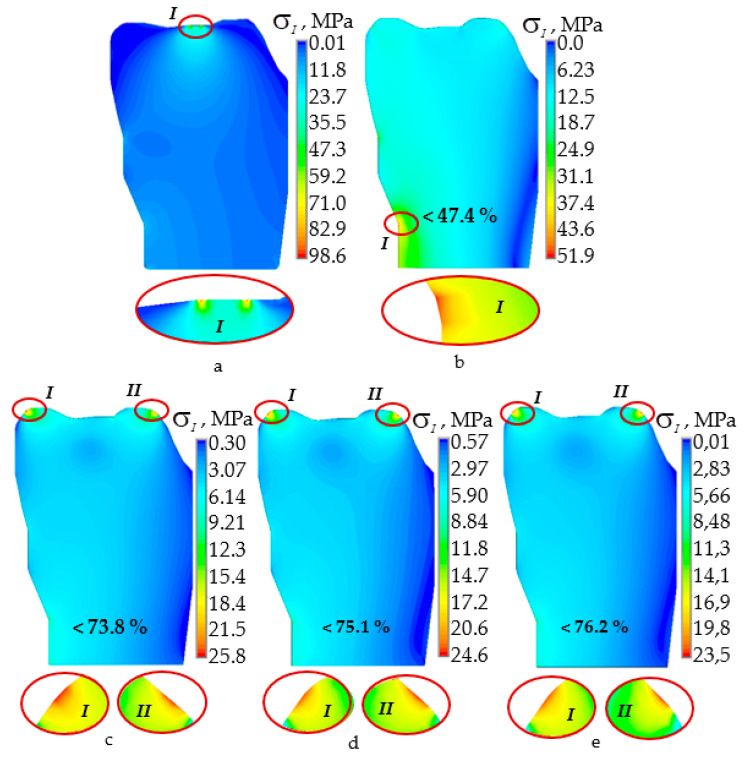
The stress intensity in the lower dentition tooth at 250 N: (**a**) case a; (**b**) case b; (**c**) case c-A; (**d**) case c-B; (**e**) case c-C; I, II—zone of maximum stress intensity.

Figure 3 and Figure 4 show the stress intensity distributions in the hard tissues of the teeth with an indentation force of 250 N for the upper and lower teeth. As expected, the use of a mouthguard leads to a significant decrease in the level of stress intensity.

In the case of teeth contact without a mouthguard, the maximal stress intensity was observed near the occlusion region. When using mouthguards, a significant decrease in the level of stress intensity was observed. In most cases, the maximum stresses are distributed over a larger area. For the multilayer EVA case, the mouthguard shifted the zone of maximum stresses towards the neck of the tooth.

For multilayer mouthguards with an A-silicon interlayer, the maximum stress intensity was observed in the contact zones, but removed from the tooth occlusion zone. When using individual mouthguards, the intensity of stresses in the tooth of the upper dentition decreased by 3.6 times when using a multilayer EVA mouthguard and on average by 6.2 times when using multilayer mouthguards with an A-silicon interlayer.

The greatest decrease in the level of stress intensity was observed when using a mouthguard with an interlayer adjusted to the geometry of the elements of the dentition (Figure 3e), maximal stress intensity was less by more than eight times.

The decrease in the maximum level of stress intensity in the hard tissues of the tooth of the lower dentition when using a multilayer individual EVA mouthguard was found to be much less than for the tooth of the upper dentition (Figure 4). The maximum stress intensity decreased by only 1.9 times. In this case, the maximum stress intensity when using a multilayer EVA mouthguard was localized near the edge of the contact area with the mouthguard.

**Figure 5:**
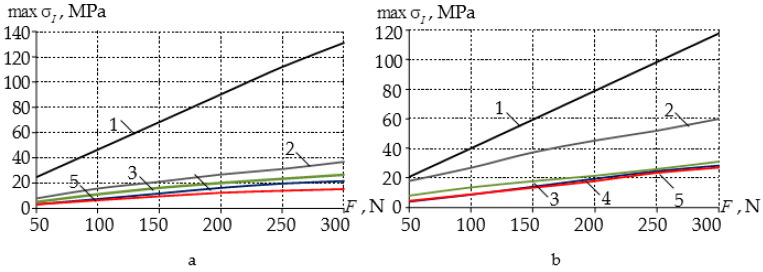
Dependence of maximal stress intensity on indentation force for the teeth of the upper (**a**) and lower (**b**) dentition: 1—case a; 2—case b; 3—case c-A; 4—case c-B; 5—case c-C.

When using multilayer mouthguards in the tooth of the lower dentition, the greatest decrease in the maximum level of stress intensity was observed on average four times more without their localization near the contact surface. Of particular interest is the analysis of the influence of the geometric characteristics of the mouthguard on the deformation behavior of the elements of the dentition. Within the framework of a series of numerical experiments, the dependences of the maximum level of stress intensity on the force of indentation in hard tissues of teeth were established for all variants of design schemes (Figure 5). As expected, the dependence of the maximum level of stress intensity on the indentation force between a pair of antagonist teeth without mouthguard usage was linear.

When using a mouthguard, the dependence of the maximal stress intensity on the indentation force was found to be close to linear. With an increase in the force of indentation, an increase in the effect of reducing the intensity of stresses in the hard tissues of the teeth was observed when using all types of mouthguards. In the tooth of the upper dentition, the greatest decrease in the level of stress intensity was observed when using a mouthguard with an interlayer adjusted to the geometry of the elements of the dentition.

**Figure 6:**
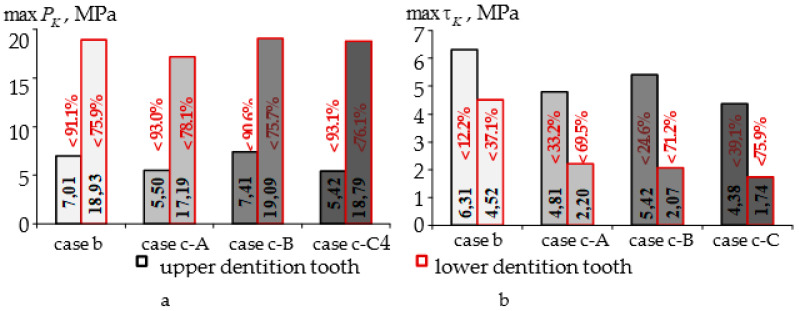
Maximal values of contact parameters: (**a**) max P_K_; (**b**) max τ_K_.

The mouthguard use was shown to have a significant impact on the parameters of contact interaction. With the contact of antagonist teeth without a mouthguard, the maximum level of contact pressure and contact shear stress reached 78.6 and 7.19 MPa with an indentation force of 250 N. Figure 6 shows a comparison of the maximum level of the parameters of the contact zones when using individual dental mouthguards of different geometric configurations.

A significant decrease in the maximum level of contact pressure can be noted for all types of dental mouthguards. The maximum level of contact shear stress decreased less than the contact pressure in the zone of contact with the tooth of the upper dentition.

The maximum contact pressure was higher in the tooth of the lower dentition, and the maximum contact shear stress was in the tooth of the upper dentition (Figure 6). As in the case of stress intensity, the maximum decrease in the level of contact interaction parameters was observed when using a mouthguard with an interlayer adjusted to the geometry of the elements of the dentition (case c-C).

The dependence of the level of plastic deformations in the mouthguard was not linear and did not exceed 30% at a maximum load.

## 4. Discussion

### 4.1. Limitation Statement

Within the framework of the study, the models of materials and objects of the study had a number of limitations.

When modeling the EVA behavior model, it is possible to consider the viscosity of the material. Additional studies of the deformation material and further clarification of the constitutive relations are required. Friction coefficients of EVA-tooth material and EVA-A-silicone are constant at 0.3; the coefficient of friction was taken from the reference literature. Investigation of the friction of materials requires specialized equipment and an original test procedure. Considering the experimentally obtained frictional properties of materials will make it possible to obtain a better picture of deformation of the elements of the dentition.

Within the framework of the task, the multilayered teeth were not considered, which introduces an additional error into the model. The tooth is a composite structure. In further studies, it is planned to consider the multilayered tooth with different properties of the materials of the layers.

The 2D FEA problem was solved. The contact between the layers of the mouthguard was considered, but the level of the parameters of the EVA-A-silicon contact zone was a lower order of magnitude than in the occlusion area. No delamination of the interlayer was observed during the simulation. Frictional contact with a previously unknown contact area, and the nature of the distribution of contact state status zones was realized in the zone of teeth closing.

The study of the influence of the geometry of the mouthguards on the deformation of the elements of the dentition was carried out in the first approximation. Researchers have a number of challenges that follow:

- Clarification of the physical, mechanical and frictional properties of the materials of the biomechanical unit;
- Clarification of the models of teeth and analysis of the influence of their multilayerness and the nature of the conjugation of layers on the deformation of the biomechanical unit in flat and axisymmetric formulations;
- Clarification of the level and type of loads acting on the biomechanical unit;
- Transition to three-dimensional models.

Mouthguard thickness has a significant impact on the patient’s comfort. Recent studies considered an influence of the mouthguard’s thickness on its performance [22,23,24]. Westerman et al. [22] revealed that the rational thickness for an EVA mouthguard is 4 mm. When a mouthguard’s thickness exceeds 4 mm, there are some negative effects on the patient’s speech and breath. Bochnig et al. [23] studied various mouthguards with thicknesses ranging from 2 up to 11 mm. It was concluded that thickness increase by insertion of additional layers results in protective properties of the construction. Sarac et al. [24] obtained similar results.

We studied multilayer mouthguards with 7 mm thickness, including harder intermediate layers. The thickness of the mouthguard can have a number of limitations when athletes use them.

### 4.2. Main Results

Almost all studies in the field of sports dental medicine pay attention to the need to use mouthguards to prevent dentofacial injuries during sport activities with a high and moderate level of injury [17,25,26]. The effectiveness of mouthguards has been proven in practice. The choice of mouthguards is wide [26]: from standard to individual, from single-layer to multilayer, from ordinary to specialized, etc. Standard and thermoplastic designs of protective mouthguards have a number of disadvantages [18]:

1. The loose fit, which leads to the need to hold the mouthguard, difficulty breathing and distorted speech.
2. Adaptation to the dentition through heating and seating is similar to taking a dental impression; this mouthguard fit on the teeth depends on the human factor, which is often not ideal.
3. Fracture and creep can be observed with repeated heat treatment, as well as an increase in the hardness of materials, which leads to its early failure and a negative effect on the athlete’s body.

The most effective protective dental constructions are individual mouthguards [18,27]. Sousa et al. [27] consider different designs of mouthguards, highlighting individual mouthguards as more effective, including multilayer ones. The classic single-layer mouthguard design reduces the maximum stress intensity in the teeth by a maximum of 54.4% [14]. The multilayer individual EVA mouthguard reduces the maximum stress intensity in teeth by 72.2%, as shown in this work. The efficiency of the multilayer mouthguard is more than 17% higher than that of the classic design. The use of a multilayer mouthguard from relatively soft material changed the stresses distribution nature in the dentition elements (Figure 3 and Figure 4b). In this case, the maximum stress level was observed in the tooth neck. The stress in the teeth decreased in general but increased in the tooth neck by 2.2–2.3 times.

Rationalization of mouthguard designs has been ongoing. The work is aimed at a number of factors [28,29]: materials, production methods, and geometry, etc. The assumption is considered that by introducing an intermediate layer made of a harder material into a structure, it is possible to achieve a better effect in protecting the teeth [16,29,30]. The intermediate interlayer of A-silicone in mouthguards made it possible to maximally reduce the stress intensity of the teeth by 87.6%, as shown in this work. Nevertheless, the insertion of additional elements or layers into the structure of the mouthguards is not always effective. For example, the mouthguard with an interlayer of a silica-nylon mesh did not show an improvement in the mechanical reaction of teeth [28]. Mouthguards with A-silicone interlayer reduced tooth stress significantly more than adaptive and multilayer EVA mouthguards, the study showed. At the same time, it was established that the interlayer geometry significantly affects the performance of the biomechanical unit. The maximum stress concentration in the upper tooth was observed, with an interlayer thickness of 22.8–27.1% of the total mouthguard thickness, but not in the teeth closing zone. The geometry of the A-silicone interlayer of the mouthguard affects the deformation of the elements of the dentition. An improperly chosen interlayer shape can lead to a stress concentration in the tooth, with further cracking.

Another feature of this work is the use of a nonlinear model of EVA material behavior. Kerr et al. [17] revealed that when choosing and analyzing the operation of mouthguards, it is necessary to consider the physical and mechanical properties of the materials from which they are made. One of the most common materials in mouthguards is the EVA polymer [28]. Currently, there is a significant amount of research devoted to the analysis of the properties of EVA from different manufacturers [29,30]. The behavior EVA model is nonlinear and reflects elastoplastic deformation. Many works consider the EVA material as elastic, for example, Lokhov et al. [16]. Consideration of EVA in terms of elastic deformation distorts the research results. The many effects and patterns were made noticeable due to the nonlinear description of the EVA operation within this study.

### 4.3. The Mouthguard Thickness Analysis

**Figure 7:**
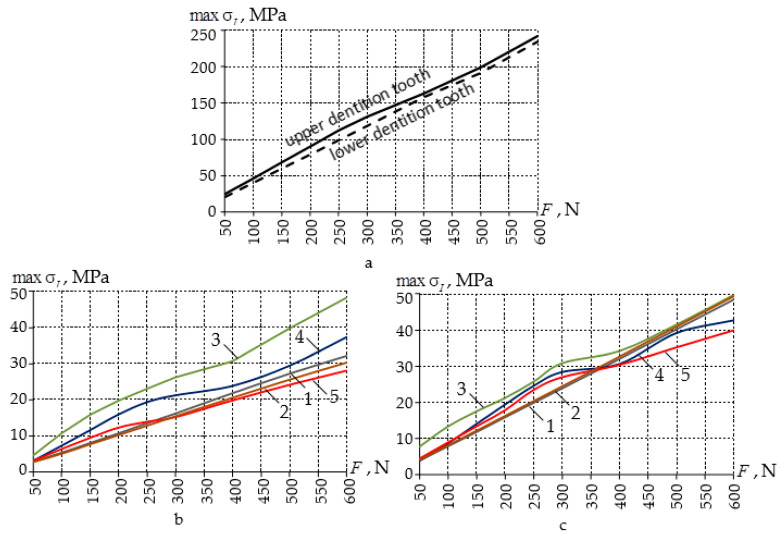
Dependence of maximal stress intensity on indentation force without (**a**) and with (**b**,**c**) a mouthguard (for teeth of the upper (**b**) and lower (**c**) teeth dentition): 1—case b of 4 mm thickness; 2—case b of 3 mm thickness; 3—case c-A; 4—case c-B; 5—case c-C.

Standard thickness of EVA mouthguards is 3–4 mm. This work considered a multilayer EVA kappa with a thickness of 7 mm. Of interest is the comparative analysis of the operation of EVA mouthguards of standard thickness and mouthguards with a layer of A-silicone. The stresses in the teeth in contact with EVA mouthguards of 3 and 4 mm thickness of a load of up to 600 N are shown in Figure 7.

It was found that with a standard thickness, the EVA aligner reduces the intensity of stresses in the teeth to a level close to the aligners with a layer of A-silicone. The maximum intensity of stresses in a mouthguard made of EVA with a thickness of 3–4 mm were observed in the neck of the tooth.

At loads less than 250–350 N, EVA splints perform better than multilayer splints with an A-silicone interlayer. Under heavy loads, the mouthguard with an interlayer adjusted to the geometry of the elements of the dentition (case c-C) reduces tooth stress better than all other aligners reviewed.

The geometry of the A-silicone interlayer has a significant effect on the deformation distributions.

## 5. Conclusions

A comparative analysis was carried out on the deformation behavior of a pair of antagonist teeth during contact with and without mouthguards of different geometric configurations, with a range of functional loads (50–300 N). Data were obtained on the intensity of stresses and deformations, the parameters of the contact zone, as well as the dependence of the maximum level of deformation characteristics on the physiological load.

Analysis of the results of a series of numerical experiments established the following conclusions:

- Mouthguards, with an additional intermediate layer of A-silicone, make it possible to reduce the level of stress intensity in the hard tissues of the teeth by 15–25% more than when using individual multilayer EVA mouthguards.
- There is no pronounced localization of zones of maximum stress intensity in the hard tissues of the teeth, for all considered options for the geometry of the intermediate layer of A-silicone, when using multilayer mouthguards with an A-silicon interlayer.
- The geometric configuration of the A-silicone interlayer has a significant effect on the stress–strain state of a pair of antagonist teeth and a mouthguard.
- The greatest decrease in the level of deformation characteristics of the investigated unit is observed when using a mouthguard with an interlayer, adjusted to the geometry of the elements of the dentition.

The thickness of 7 mm multilayer mouthguards can lead to a number of limitations when used by athletes. Additional practical research is required on the physical and psychoemotional state of patients when they are using it.

## Data Availability

Not applicable.
